# Silica Nanoparticles Shed Light on Intriguing Cellular Pathways in Human Tracheal Smooth Muscle Cells: Revealing COX-2/PGE_2_ Production through the EGFR/Pyk2 Signaling Axis

**DOI:** 10.3390/biomedicines12010107

**Published:** 2024-01-04

**Authors:** Wen-Bin Wu, I-Ta Lee, Yan-Jyun Lin, Ssu-Ying Wang, Li-Der Hsiao, Chuen-Mao Yang

**Affiliations:** 1 School of Medicine, Fu Jen Catholic University, New Taipei City 242062, Taiwan; wenbin@mail.fju.edu.tw; 2 Graduate Institute of Biomedical and Pharmaceutical Science, Fu Jen Catholic University, New Taipei City 242062, Taiwan; lily00134009@gmail.com (S.-Y.W.); lidesiao@livemail.tw (L.-D.H.); 3 School of Dentistry, College of Oral Medicine, Taipei Medical University, Taipei 110301, Taiwan; itlee0128@tmu.edu.tw; 4 Institute of Translational Medicine and New Drug Development, College of Medicine, China Medical University, Taichung 406040, Taiwan; u108306002@cmu.edu.tw

**Keywords:** airway, cyclooxygenase-2, prostaglandin, silica oxide, nanoparticles

## Abstract

The use of manufactured silica nanoparticles (SiNPs) has become widespread in everyday life, household products, and various industrial applications. While the harmful effects of crystalline silica on the lungs, known as silicosis or chronic pulmonary diseases, are well understood, the impact of SiNPs on the airway is not fully explored. This study aimed to investigate the potential effects of SiNPs on human tracheal smooth muscle cells (HTSMCs). Our findings revealed that SiNPs induced the expression of cyclooxygenase-2 (COX-2) mRNA/protein and the production of prostaglandin E_2_ (PGE_2_) without causing cytotoxicity. This induction was transcription-dependent, as confirmed by cell viability assays and COX-2 luciferase reporter assays. Further analysis, including Western blot with pharmacological inhibitors and siRNA interference, showed the involvement of receptor tyrosine kinase (RTK) EGF receptor (EGFR), non-RTK Pyk2, protein kinase Cα (PKCα), and p42/p44 MAPK in the induction process. Notably, EGFR activation initiated cellular signaling that led to NF-κB p65 phosphorylation and translocation into the cell nucleus, where it bound and stimulated COX-2 gene transcription. The resulting COX-2 protein triggered PGE_2_ production and secretion into the extracellular space. Our study demonstrated that SiNPs mediate COX-2 up-regulation and PGE_2_ secretion in HTSMCs through the sequential activation of the EGFR/Pyk2/PKCα/p42/p44MAPKs-dependent NF-κB signaling pathway. Since PGE_2_ can have both physiological bronchodilatory and anti-inflammatory effects, as well as pathological pro-inflammatory effects, the increased PGE_2_ production in the airway might act as a protective compensatory mechanism and/or a contributing factor during airway exposure to SiNPs.

## 1. Introduction

Exposure to environmental toxicants poses significant health risks, especially concerning pulmonary inflammation [1]. Indoor sources like cigarette smoke, mycotoxins, and airborne particles such as asbestos, silica, and heavy metals contribute to this issue. Chronic exposure, particularly from cigarette smoke, can lead to chronic obstructive pulmonary disease (COPD), a major global cause of death [2,3].

Silica, present in crystalline and amorphous forms, comprises silicon dioxide (SiO_2_) and is abundant in the earth’s crust. While natural amorphous silica is generally non-toxic, exposure to crystalline silica can cause lung conditions like silicosis, emphysema, chronic bronchitis, or COPD [4]. Manufactured silica nanoparticles (SiNPs) are widely used in everyday products and industrial applications like paints, rubber, toothpaste, silicones, inks, and cosmetics [5]. Recent studies suggest that manufactured amorphous SiNPs may exhibit toxicity similar to micrometric crystalline silica [6]. Consequently, concerns are growing about the non-tumoral lung effects of exposure to crystalline and amorphous silica at micro- and nanometric scales [4].

Cyclooxygenase (COX), also known as prostaglandin H/G synthase, serves as the crucial enzyme responsible for generating prostanoids from arachidonic acid (AA), which is hydrolyzed from cell membrane phospholipids through the action of phospholipase A_2_ [7]. The term “prostanoid” encompasses a group of molecules, including prostaglandins (PGs), prostacyclins, and thromboxanes. There exist two distinct isoforms of COX: COX-1 and COX-2, each encoded by different genes (official gene symbols: PTGS1 and PTGS2, respectively) [8]. COX-1 is generally considered a constitutively expressed “housekeeping” enzyme, whereas COX-2 can be either inducible or constitutive, contingent upon the specific tissue context [7,8]. COX-2, as an inflammation-associated enzyme, can be induced by a variety of cytokines and inflammatory mediators found in different inflammatory cells [9]. In states of both acute and chronic inflammation, the induced COX-2 takes on a primary role in synthesizing prostanoids [8]. These prostanoid molecules significantly contribute to the pathological processes observed in various inflammatory conditions, including but not limited to cancer, rheumatoid arthritis, Alzheimer’s disease, and respiratory disorders [7]. The induction of COX-2 and subsequent prostanoid synthesis play pivotal roles in the progression and exacerbation of these inflammatory states, making COX-2 an attractive target for therapeutic interventions aimed at mitigating inflammation-related diseases [9]. Understanding the intricate regulatory mechanisms and roles of COX isoforms is essential in unraveling the complexities of inflammatory disorders and developing effective treatments for these conditions.

Prostaglandin E_2_ (PGE_2_) is a significant lipid mediator produced from AA under the catalytic action of COX, a pivotal rate-limiting enzyme in its synthesis [10]. PGE_2_ exerts its wide-ranging effects by binding to four distinct receptor subtypes (EP1–EP4), influencing crucial physiological processes such as temperature regulation (pyrexia), pain perception, and inflammatory responses [10,11]. Under normal circumstances, PGE_2_ plays a crucial role in immune responses, manifesting bronchodilatory and anti-inflammatory effects through the activation of EP2 and/or EP4 receptors [11]. However, in pathological conditions like COPD, there is an abnormal elevation in PGE_2_ levels, which contributes to intensified inflammation and angiogenesis within the airways [12]. In essence, PGE_2_, regulated by COX activity and receptor interactions, acts as a potent modulator of various physiological responses [13]. Its dysregulation, particularly in conditions like COPD, underscores the intricate balance required for maintaining normal immune and inflammatory processes. Understanding these complexities is crucial for unraveling the mechanisms underlying inflammatory disorders and developing targeted therapeutic strategies to restore the balance and alleviate associated pathological symptoms.

Exposure to silica induces oxidative stress in human cells, prompting adaptive responses [14]. Notably, metal oxide nanoparticles like SiO_2_ and titanium dioxide (TiO_2_) can penetrate bronchial epithelial barriers, with smaller, negatively charged particles exhibiting higher translocation rates [15]. Additionally, SiNPs used in drug delivery systems can infiltrate the central nervous system, leading to neurotoxicity [16]. Oxidative stress, characterized by an imbalance between reactive oxygen species (ROS) production and antioxidant defense, triggers aberrant activation of intracellular signaling pathways involving various kinases and transcription factors [17]. This activation induces COX-2 expression, leading to the production of prostaglandins, contributing to tissue inflammation [18]. In the context of respiratory health, the widespread use of SiNPs in industry raises concerns regarding their impact on the human respiratory system, especially on bronchial smooth muscle cells responsible for bronchial contractility. To address this concern, this study delved into the effects of SiNPs on COX-2 expression, prostaglandin release, and cellular signaling in human tracheal smooth muscle cells (HTSMCs).

In summary, understanding the intricate mechanisms by which SiNPs influence cellular signaling pathways and exacerbate oxidative stress is vital for comprehending their potential impact on respiratory health. The investigation into COX-2 expression and prostaglandin release in tracheal smooth muscle cells provides valuable insights into the specific cellular responses triggered by SiNPs. This knowledge is crucial for developing targeted interventions and preventive strategies, safeguarding individuals from the adverse effects of silica nanoparticle exposure on the respiratory system.

## 2. Materials and Methods

### 2.1. Materials

SiNPs were acquired from Sigma-Aldrich, based in St. Louis, MO, USA. These were in nanopowder form, characterized by a particle size ranging from 10–20 nm and possessing a purity level of 99.5% on a trace metals basis (catalog number: 637238). Essential laboratory materials including fetal bovine serum (FBS) and TRIzol reagent were procured from Invitrogen, located in Carlsbad, CA, USA. Additionally, Hybond C membrane and enhanced chemiluminescence (ECL) reagents were sourced from GE Healthcare Biosciences, based in Buckinghamshire, England, UK. Specific biochemicals, namely actinomycin D (Act. D), cycloheximide (CHI), as well as inhibitors such as U0126, PF431396, Gö6976, AG1478, and Bay117082, were obtained from Biomol, Plymouth Meeting, PA, USA. Various antibodies, including anti-COX-2 (#12282), anti-phospho-EGFR (Tyr1173, #4407), anti-phospho-Pyk2 (Tyr402, #3291), anti-phospho-PKCα (Thr638/641, #9375), anti-phospho-p42/p44 MAPK (Thr202/Tyr204, #9101), and anti-phospho-NF-κB p65 (Ser536, #3031), were supplied by Cell Signaling Technology, Danvers, MA, USA. Santa Cruz, located in Santa Cruz, CA, USA, provided anti-EGFR (sc-373746), anti-p42/p44 MAPK (sc-7383), and anti-NF-κB p65 (sc-8008) antibodies. Anti-Pyk2 (#ab32448) was procured from Abcam, Cambridge, UK. Detection antibodies, peroxidase AffiniPure goat anti-Rabbit IgG (H+L) (#111035003), and peroxidase AffiniPure goat anti-Mouse IgG (H+L) (#115035003) were purchased from Jackson, West Grove, PA, USA. The anti-GAPDH antibody was sourced from Biogenesis, based in Bournemouth, UK. The quantification of protein levels was conducted using the Bicinchoninic Acid (BCA) protein assay kit from Pierce, located in Rockford, IL, USA. Enzymes and other necessary chemicals were obtained from Sigma, headquartered in St. Louis, MO, USA.

### 2.2. HTSMCs Culture

HTSMCs were procured from ScienCell Research Laboratories, situated in San Diego, CA, USA. These cells were derived from the human trachea, isolated and cryopreserved at the first passage, and delivered while frozen. Each vial contained more than 5 × 10^5^ cells in a 1 mL volume. The cells were cultivated in Dulbecco’s Modified Eagle Medium/Nutrient Mixture F-12 (DMEM/F-12) supplemented with 10% FBS, 2 mM glutamine, and antibiotics (100 U/mL of penicillin G, 100 μg/mL of streptomycin, and 250 ng/mL of fungizone) at 37 °C in a humidified atmosphere with 5% CO_2_. Upon reaching confluence (typically within 4 days), the cells were treated with 0.05% (*w/v*) trypsin/0.53 mM EDTA solution for 1 min at 37 °C to detach them. The resulting cell suspension was then diluted with DMEM/F-12 medium containing 10% FBS and 2 mM glutamine. For experimental purposes related to protein expression and mRNA accumulation, the cell suspension was distributed into 12-well culture plates (1 mL per well) and 6-well culture plates (2 mL per well). All experiments were conducted using cells from passages 4 to 7.

### 2.3. Western Blot

Cells that had been arrested in growth were exposed to varying concentrations of SiNPs at 37 °C for specified time intervals. In cases where pharmacological inhibitors were employed, they were pre-treated for 1 h prior to SiNPs exposure. Following the incubation period, cells were swiftly washed, harvested, and denatured by heating for 15 min at 95 °C. The denatured samples were then centrifuged at 45,000× *g* at 4 °C to prepare whole cell extracts. These samples underwent SDS-PAGE using a 10% running gel and were subsequently transferred onto nitrocellulose membranes. The membranes were sequentially incubated overnight at 4 °C with specific primary antibodies. After this, they were treated with a 1:2000 dilution of either anti-rabbit or anti-mouse antibody for 1 h at room temperature. Post-incubation, extensive washing with TTBS (Tris-buffered saline with Tween 20) was carried out. Immunoreactive bands were visualized using an enhanced chemiluminescence reagent. The resulting immunoblot images were captured using a UVP BioSpectrum 500 imaging system located in Upland, CA, USA. Densitometry analysis of the bands was performed using UN-SCAN-IT gel software based in Orem, UT, USA.

### 2.4. Real-Time PCR

For real-time PCR analysis, total RNA was extracted from SiNPs-treated HTSMCs cultivated in 6-well culture plates over specified time intervals using 500 μL TRIzol. The RNA concentration was determined spectrophotometrically at 260 nm/280 nm. Following established protocols [19], 5 μg of total RNA was reverse-transcribed into cDNA, which was subsequently used as a template for PCR amplification. Specific primers and probe mixtures were employed for COX-2 and GAPDH genes. PCR reactions were conducted using the StepOnePlus™ Real-Time PCR System (Applied Biosystems™/Thermo Fisher Scientific, Foster City, CA, USA). The relative abundance of the target gene was calculated using the formula 2^(Ct test gene − Ct GAPDH)^ (where Ct represents the threshold cycle).

### 2.5. Measurement of PGE_2_ Release

To determine the PGE_2_ levels in HTSMCs after SiNPs treatment, the concentration of PGE_2_ in the cell culture medium was quantified utilizing an Enzo Life Sciences PGE_2_ ELISA kit based in Farmingdale, NY, USA. The analysis was conducted following the guidelines outlined in the product manual.

### 2.6. Transient Transfection with siRNAs

In the experimental setup, HTSMCs at a concentration of 2 × 10^5^ cells/mL were cultured in 12-well plates for 5 days until they reached around 90% confluence. Following a single wash with PBS, each well received 0.5 mL of serum-free DMEM/F-12 medium. Specific siRNAs were employed: EGFR siRNA (SASI_Hs01_00215449; NM_005228) was procured from Dharmacon, Inc. (Lafayette, CO, USA), and Pyk2 (SASI_Hs01_00032249; NM_004103), PKCα (SASI_Hs01_00018816), p42 (SASI_Hs01_00124656), p44 (SASI_Hs01_00153005), p65 (SASI_Hs01_00171090), and scramble siRNA were sourced from Sigma-Aldrich (St. Louis, MO, USA). For transient transfection, siRNAs were prepared at a final concentration of 100 nM using Lipofectamine 2000 reagent siRNA transfection reagent, following the manufacturer’s instructions (Carlsbad, CA, USA). These prepared siRNA solutions were then directly added to the cells, aligning with the methodology previously described [20].

### 2.7. Promoter Assay

The human COX-2 promoter region spanning from −483 to +37 was inserted into the pGL3-basic vector, which carries the luciferase reporter system. Specific nucleotides within this region were substituted, indicated by underlined bases. All plasmids were prepared using QIAGEN plasmid DNA preparation kits. These constructs were introduced into HTSMCs through transfection utilizing Lipofectamine 2000 reagent, following the manufacturer’s guidelines. Following exposure to SiNPs, cells were harvested and mechanically disrupted through sonication in a lysis buffer (containing 25 mM Tris, pH 7.8, 2 mM EDTA, 1% Triton X-100, and 10% glycerol). After centrifugation, samples of the supernatants were utilized to assess promoter activity via a luciferase assay system obtained from Promega, Madison, WI, USA. Firefly luciferase activities were normalized based on β-galactosidase activity.

### 2.8. Chromatin Immunoprecipitation (ChIP) Assay

Soluble chromatin was immunoprecipitated using an anti-p65 antibody. After thorough washing and elution, the precipitates were subjected to overnight heating at 65 °C to reverse the DNA-protein cross-linking process. Briefly, HTSMCs were fixed with 1% formaldehyde for 30 min at room temperature and quenched using glycine (1.25 M). They were then washed twice with ice-cold PBS, and the DNA fragments were lysed using ice-cold PBS. The purified DNA was subsequently amplified via PCR, employing specific primers targeting the region containing NF-κB binding sites within the COX-2 promoter (NF-κB/kappa1): sense primer 5′-GGCAAAGACTGCGAAGAAGA-3′ and antisense primer 5′-AAAATCGGAAACCCAGGAAG-3′. The resulting PCR fragments were analyzed using semi-quantitative PCR on a 2% agarose gel in 1X TAE buffer containing ethidium bromide or quantitative PCR with SYBR Green.

### 2.9. Cell Viability

To assess cell viability, cells were seeded in 12-well plates and allowed to adhere overnight in DMEM/F-12 medium containing 10% FBS. Subsequently, the cells were exposed to varying concentrations of SiNPs (0, 25, 50, and 100 μg/mL) for a specified duration (16 h). Cell viability was evaluated using the Cell Counting Kit-8, which utilizes the highly water-soluble tetrazolium salt WST-8 [2-(2-methoxy-4-nitrophenyl)-3-(4-nitrophenyl)-5-(2,4-disulfophenyl)-2H-tetrazolium, monosodium salt]. This salt produces a water-soluble formazan dye upon reduction in the presence of an electron carrier.

### 2.10. Statistical Analysis

The data are displayed as the average value ± standard error of the mean (SEM), based on three independent experiments (*n* = 3, from distinct cell culture preparations). For statistical analysis, we utilized GraphPad Prism Program version 6.0 (GraphPad, San Diego, CA, USA), adhering to the method outlined in reference [19]. We employed a one-way analysis of variance (ANOVA), followed by Tukey’s post hoc test for further analysis. A *p*-value threshold of 0.05 was set for statistical significance. In cases where error bars were smaller than the symbols used in the graphs, they were not included in the display.

## 3. Results

### 3.1. SiNPs Induces COX-2 Expression and PGE_2_ Synthesis in HTSMCs

**Figure 1:**
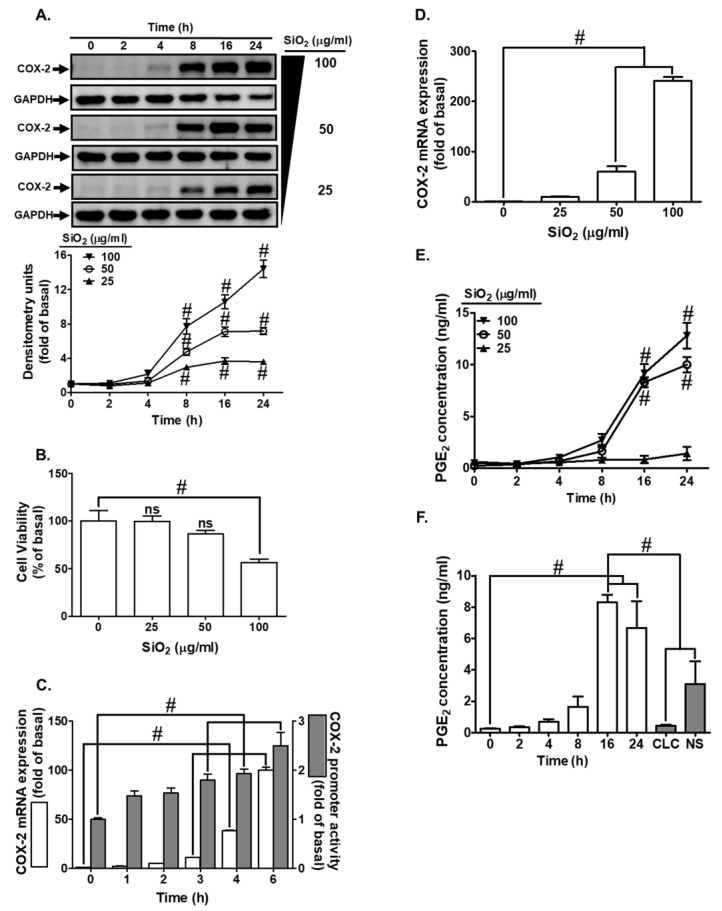
Time- and concentration-dependent patterns of SiNPs-induced COX-2 expression and PGE_2_ synthesis in HTSMCs. (**A**) COX-2 expression in HTSMCs was induced by treating them with different concentrations (0, 25, 50, and 100 μg/mL) of SiNPs at various time points (0, 2, 4, 8, 16, and 24 h). Western blot was used to analyze COX-2 expression, with GAPDH serving as an internal control. (**B**) Cell viability was assessed in the presence or absence of SiNPs using the Cell Count Kit-8 assay. (**C**) HTSMCs were treated with SiNPs (50 μg/mL) for specified time intervals (0, 1, 2, 3, 4, and 6 h), and total RNA was extracted. Real-time PCR was performed to measure COX-2 and GAPDH mRNA expression levels. COX-2 promoter activity was determined using promoter assay. (**D**) Total RNA was extracted from cells treated with different concentrations of SiNPs (0, 25, 50, and 100 μg/mL) for 4 h. (**E**) PGE_2_ synthesis in HTSMCs was induced by treating them with various concentrations (0, 25, 50, and 100 μg/mL) of SiNPs at different time points (0, 2, 4, 8, 16, and 24 h). (**F**) Cells were exposed to SiNPs (50 μg/mL) for specified time intervals or pre-treated with celecoxib (CLC, 3 μM) or NS-398 (1 μM) for 1 h, followed by SiNPs (50 μg/mL) incubation for 16 h. The medium was collected and analyzed for PGE_2_ secretion. The data are represented as mean ± S.E.M. from three separate experiments. # *p* < 0.05 indicates a significant difference between the groups. “ns” indicates not significant.

In our study, we investigated the impact of SiNPs on COX-2 expression in HTSMCs. Cells were exposed to varying concentrations of SiNPs (25, 50, and 100 μg/mL) for different durations (0, 2, 4, 8, 16, and 24 h). Our findings revealed that SiNPs induced COX-2 protein expression in a time- and concentration-dependent manner, with significant up-regulation observed at 8 h, 16 h, and 24 h (Figure 1A). To assess SiNPs’ cytotoxic effects, cells were treated with SiNPs at concentrations of 0, 25, 50, and 100 μg/mL for 16 h. The results (Figure 1B) indicated reduced cell viability only at the highest concentration of 100 μg/mL. Further experiments employed a concentration of 50 μg/mL SiNPs, challenging HTSMCs at various time points (0, 1, 2, 3, 4, and 6 h). COX-2 mRNA expression and promoter activity were evaluated using real-time PCR and promoter assay, respectively. SiNPs induced a time-dependent increase in COX-2 mRNA accumulation, peaking significantly at 3 h, 4 h, and 6 h of treatment. Similarly, SiNPs-driven COX-2 promoter activity exhibited a time-dependent pattern, with significant enhancement observed at 3 h, 4 h, and 6 h (Figure 1C). Moreover, when cells were exposed to different SiNPs concentrations (25, 50, and 100 μg/mL) for 4 h, COX-2 mRNA expression displayed a dose-dependent response in SiNPs-treated HTSMCs (Figure 1D). To explore downstream effects of SiNPs-induced COX-2 expression, we examined PGE_2_ synthesis. Cells were treated with SiNPs at concentrations of 25, 50, and 100 μg/mL for varying durations (0, 2, 4, 8, 16, and 24 h). Consistent results revealed that SiNPs-induced PGE_2_ secretion exhibited both time- and concentration-dependent patterns (Figure 1E). Furthermore, we investigated if SiNPs could enhance PGE_2_ production via the COX-2-dependent pathway. Selective COX-2 inhibitors (celecoxib and NS-398) were employed, resulting in a significant reduction in SiNPs-induced PGE_2_ secretion in HTSMCs (Figure 1F). This indicates the pivotal role of COX-2 in mediating SiNPs-induced PGE_2_ release.

### 3.2. SiNPs Stimulate the Expression of COX-2 and the Synthesis of PGE_2_ through Both Transcriptional and Translational Control Mechanisms

**Figure 2:**
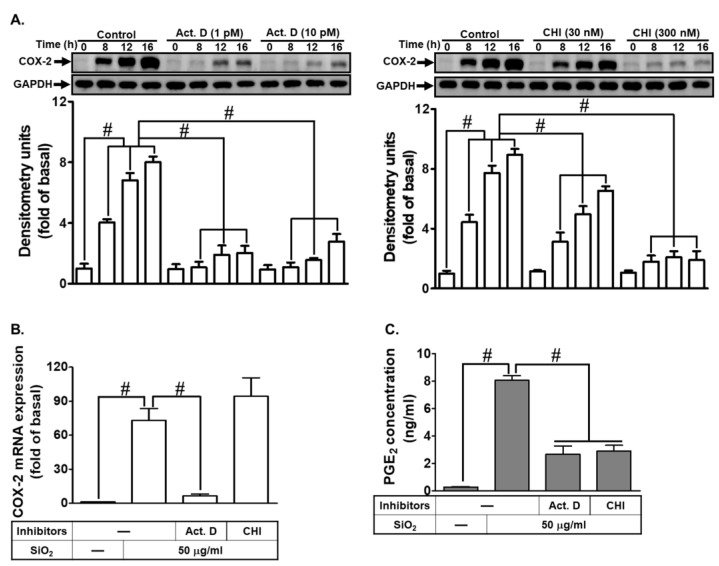
Effects of Act. D and CHI on SiNPs-induced COX-2 expression and PGE_2_ synthesis in HTSMCs. (**A**) HTSMCs were pre-incubated with Act. D (1 or 10 pM) or CHI (30 or 300 nM) for 1 h and then exposed to SiNPs (50 μg/mL) for 0, 8, 12, 16 h. Western blot was conducted to analyze COX-2 expression, with GAPDH used as an internal control. (**B**,**C**) The impact of Act. D (10 pM) or CHI (300 nM) on COX-2 mRNA expression (**B**) and PGE_2_ synthesis (**C**) induced by SiNPs (50 μg/mL) in HTSMCs. Cells were pre-treated with Act. D (10 pM) or CHI (300 nM) for 1 h and then incubated with SiNPs (50 μg/mL) for 4 h (**B**) or 16 h (**C**). The data are represented as mean ± S.E.M. from three separate experiments. # *p* < 0.05 indicates a significant difference between the groups.

In order to establish the mechanisms behind SiNPs-induced COX-2 expression and PGE_2_ secretion in HTSMCs, we employed Act. D, a transcription inhibitor; and CHI, a translation inhibitor. HTSMCs were pre-treated with either inhibitor before SiNPs’ exposure. The results revealed that both Act. D and CHI significantly reduced SiNPs-induced COX-2 protein expression (as shown in Figure 2A). Interesti ngly, at the transcriptional level, Act. D suppressed SiNPs-induced COX-2 mRNA expression, whereas CHI did not have the same effect (Figure 2B). Additionally, both Act. D and CHI treatments led to a decrease in PGE_2_ secretion (Figure 2C). These findings indicate that SiNPs-induced COX-2 expression operates through de novo mRNA synthesis and subsequent protein production, ultimately leading to PGE_2_ generation in HTSMCs.

### 3.3. SiNPs-Induced COX-2 Expression and PGE_2_ Release Are Mediated through the Activation of the EGFR Receptor Tyrosine Kinase

**Figure 3:**
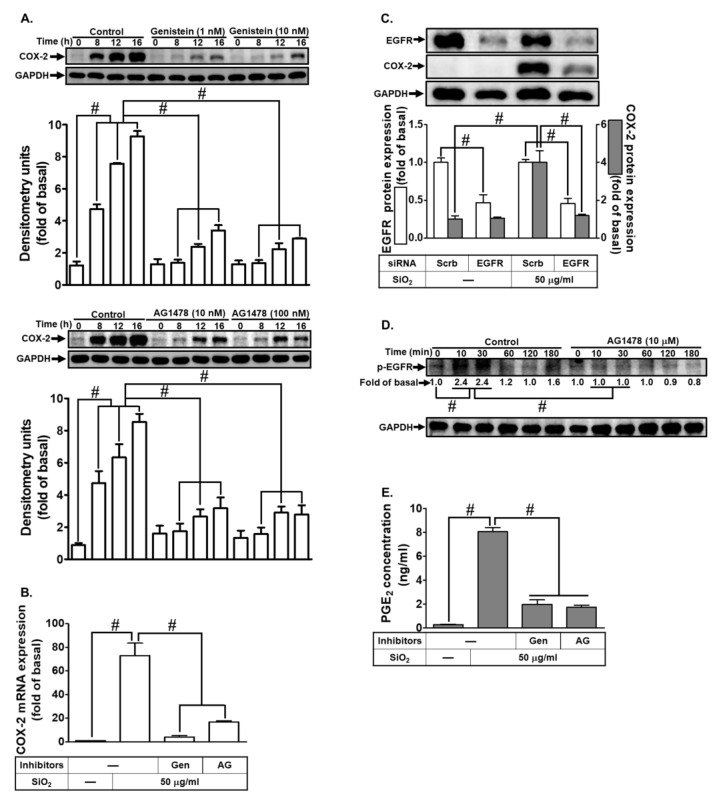
EGFR is involved in SiNPs-induced COX-2 expression and PGE_2_ synthesis in HTSMCs. (**A**) Cells were pre-incubated with genistein (0, 1, and 10 nM) or AG1478 (0, 10, and 100 nM) for 1 h and then exposed to SiNPs (50 μg/mL) for 0, 8, 12, and 16 h. Western blot was conducted to assess the expression of COX-2, with GAPDH serving as an internal control. (**B**) Cells were pre-treated with genistein (10 nM) or AG1478 (100 nM) for 1 h and subsequently incubated with SiNPs (50 μg/mL) for 4 h. Total RNA was extracted and subjected to real-time PCR analysis. (**C**) Cells were transfected with EGFR siRNA and then treated with SiNPs (50 μg/mL) for 16 h. Western blot was performed to evaluate the expression of total EGFR, COX-2, and GAPDH. (**D**) Cells were pre-treated with or without AG1478 (10 μM) for 1 h, followed by incubation with SiNPs for the specified time intervals. Western blot was used to assess the expression of phosphorylated EGFR. (**E**) Cells were pre-treated with genistein (10 nM) or AG1478 (100 nM) for 1 h and then exposed to SiNPs (50 μg/mL) for 16 h. The culture medium was collected and analyzed using ELISA to measure PGE_2_ secretion. The data are represented as mean ± S.E.M. from three separate experiments. # *p* < 0.05 indicates a significant difference between the groups.

Previous research has indicated a potential link between silica-induced inflammation and receptor tyrosine kinases [21]. Silica, a type of particulate matter (PM), and components of PM have been shown to activate EGFR signaling, triggering a pro-inflammatory response [22]. However, the specific role of EGFR in SiNPs-treated HTSMCs was not well understood. To investigate this, we utilized receptor tyrosine kinase (RTK) inhibitors, namely genistein and AG1478, to explore whether RTKs regulated SiNPs-induced COX-2 expression. Our findings demonstrated that both genistein and AG1478 effectively decreased SiNPs-induced COX-2 protein and mRNA expressions (as illustrated in Figure 3A,B). Furthermore, by down-regulating EGFR through EGFR siRNA transfection, we observed a reduction in SiNPs-induced COX-2 expression (Figure 3C). Additionally, treatment with AG1478 (10 μM) inhibited the time-dependent phosphorylation of EGFR stimulated by SiNPs in HTSMCs, providing evidence for the involvement of EGFR in SiNPs-induced COX-2 expression (Figure 3D). Moreover, the secretion of PGE_2_ was also diminished upon treatment with these two inhibitors (genistein and AG1478) (Figure 3E). These results strongly suggest that EGFR activation plays a crucial role in SiNPs-induced COX-2 expression in HTSMCs.

### 3.4. SiNPs Induce COX-2 Expression and PGE_2_ Secretion via Non-RTK Pyk2

**Figure 4:**
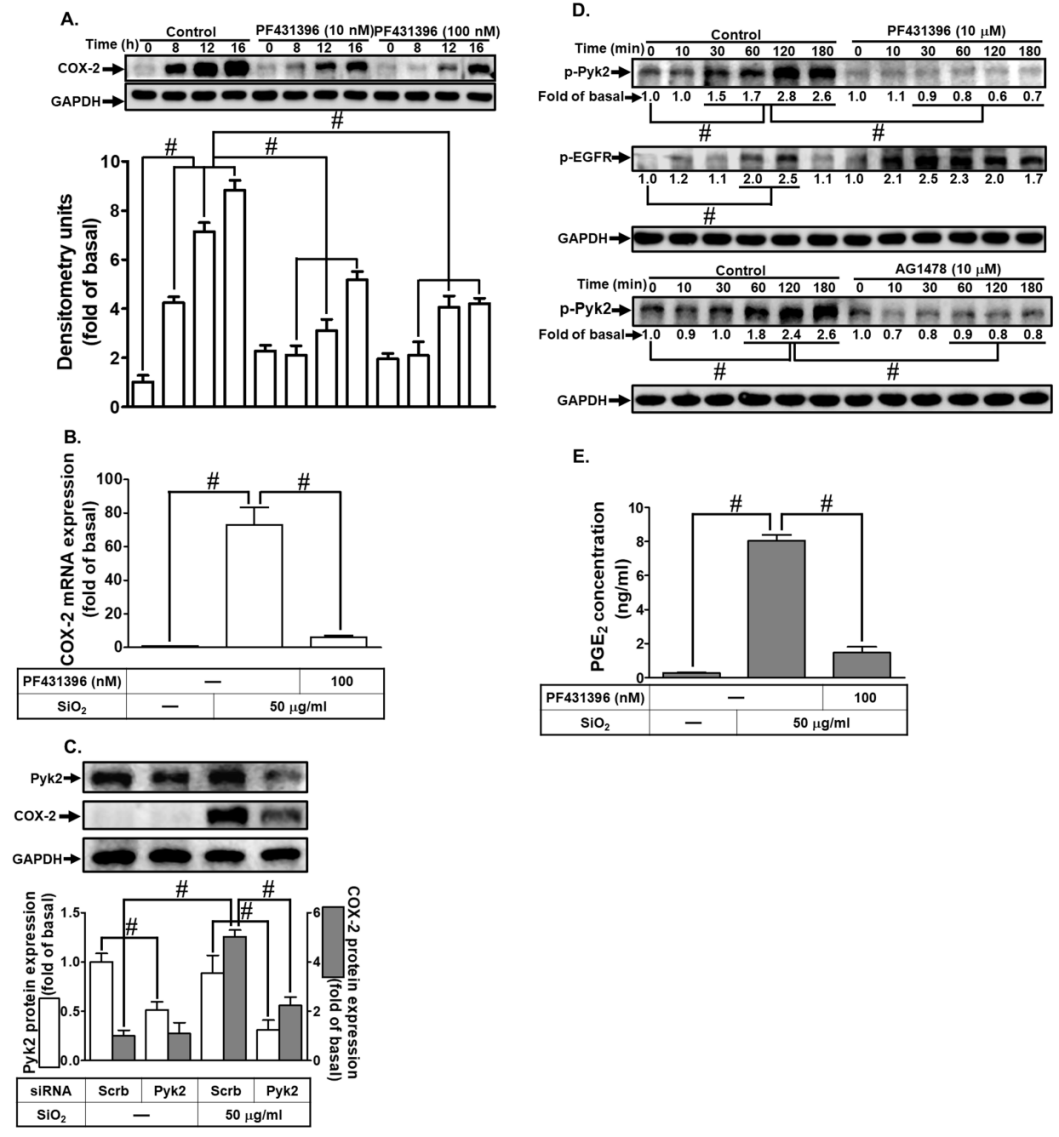
SiNPs induce COX-2 expression and PGE_2_ secretion via Pyk2 in HTSMCs. (**A**) Cells were pre-treated with PF431396 (0, 10, and 100 nM) for 1 h and then exposed to SiNPs (50 μg/mL) for 0, 8, 12, and 16 h. Western blot was conducted to assess the expression of COX-2, with GAPDH serving as an internal control. (**B**) Cells were pre-treated with PF431396 (100 nM) for 1 h and subsequently incubated with SiNPs (50 μg/mL) for 4 h. Total RNA was extracted and subjected to real-time PCR analysis. (**C**) Cells were transfected with Pyk2 siRNA and then treated with SiNPs (50 μg/mL) for 16 h. Western blot was performed to evaluate the expression of total Pyk2, COX-2, and GAPDH. (**D**) Cells were pre-treated with PF431396 (10 μM) or AG1478 (10 μM) for 1 h, followed by incubation with SiNPs for the specified time intervals. Western blot was used to assess the expression of phosphorylated Pyk2 and EGFR. (**E**) Cells were pre-treated with PF431396 (100 nM) for 1 h and then exposed to SiNPs (50 μg/mL) for 16 h. The culture medium was collected and analyzed using ELISA to measure PGE_2_ secretion. The data are represented as mean ± S.E.M. from three separate experiments. # *p* < 0.05 indicates a significant difference between the groups.

The involvement of signaling pathways mediated by both RTKs and non-RTKs has been implicated in the pathogenesis of asthma and COPD [23]. Non-RTKs, including kinases like Pyk2, have been found to play a significant role in pulmonary inflammatory diseases. For instance, Pyk2 has been shown to regulate inflammatory cell migration in vitro and modulate allergic airway inflammation, cytokine secretion, and hyperresponsiveness in a mouse model of asthma [24]. In this study, we used the Pyk2 inhibitor PF431396 to investigate the role of Pyk2 in SiNPs-induced COX-2 expression. Our results indicated that PF431396 attenuated COX-2 expression at both the protein and mRNA levels in SiNPs-treated HTSMCs (Figure 4A,B). These findings were further supported by experiments in which total Pyk2 expression was down-regulated through Pyk2 siRNA transfection, leading to a reduction in SiNPs-induced COX-2 expression (Figure 4C). Additionally, SiNPs-induced phosphorylation of Pyk2 was diminished by treatment with PF431396 or AG1478. Notably, PF431396 treatment did not inhibit SiNPs-induced EGFR phosphorylation (Figure 4D). Moreover, PF431396 treatment attenuated SiNPs-stimulated PGE_2_ secretion in HTSMCs, as demonstrated in Figure 4E. Taken together, these results suggest that Pyk2 participates in SiNPs-induced COX-2 expression via the EGFR-dependent pathway in HTSMCs.

### 3.5. Involvement of PKCα in SiNPs-Induced COX-2 Expression and PGE_2_ Synthesis

**Figure 5:**
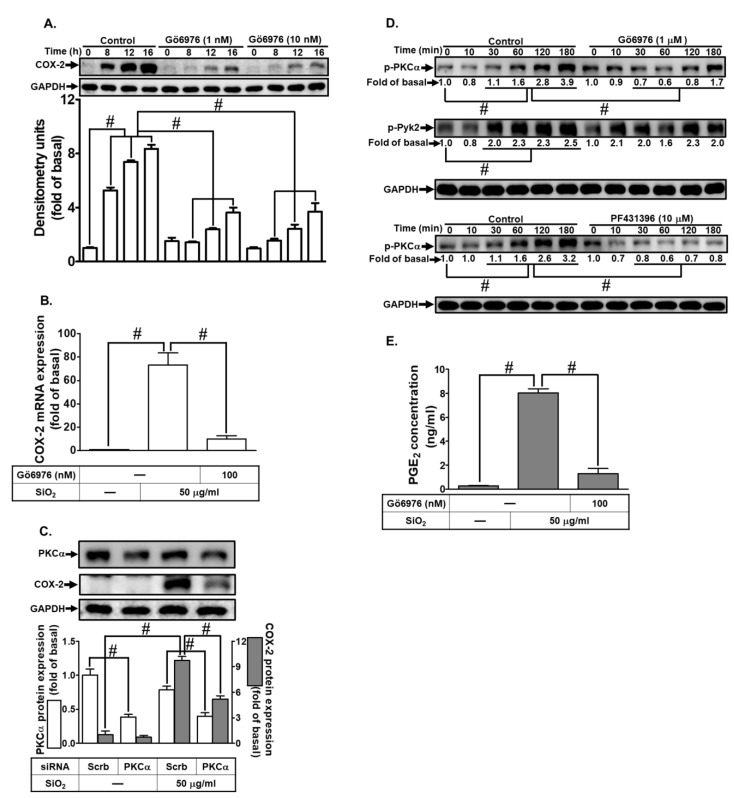
Involvement of PKCα in SiNPs-induced COX-2 expression and PGE_2_ synthesis in HTSMCs. (**A**) Cells were pre-incubated with Gö6976 (0, 1, and 10 nM) for 1 h and then exposed to SiNPs (50 μg/mL) for 0, 8, 12, and 16 h. Western blot was conducted to assess the expression of COX-2, with GAPDH serving as an internal control. (**B**) Cells were pre-treated with Gö6976 (10 nM) for 1 h and subsequently incubated with SiNPs (50 μg/mL) for 4 h. Total RNA was extracted and analyzed by real-time PCR. (**C**) Cells were transfected with PKCα siRNA and then treated with SiNPs (50 μg/mL) for 16 h. Western blot was performed to evaluate the expression of total PKCα, COX-2, and GAPDH. (**D**) Cells were pre-treated with Gö6976 (1 μM) or PF431396 (10 μM) for 1 h, followed by incubation with SiNPs for the indicated time intervals. Western blot was used to analyze the expression of phosphorylated PKCα and Pyk2. (**E**) Cells were pre-treated with Gö6976 (10 nM) for 1 h and then exposed to SiNPs (50 μg/mL) for 16 h. The culture medium was collected and analyzed using ELISA to measure PGE_2_ secretion. The data are represented as mean ± S.E.M. from three separate experiments. # *p* < 0.05 indicates a significant difference between the groups.

Given the pivotal role of PKCs in airway diseases, particularly asthma and COPD [25], we investigated their involvement in SiNPs-induced COX-2 expression in HTSMCs using the selective PKCα inhibitor, Gö6976. Treatment with Gö6976 effectively suppressed SiNPs-induced COX-2 protein and mRNA expression (Figure 5A,B). To further confirm the specific contribution of PKCα, we down-regulated PKCα protein levels through siRNA transfection in HTSMCs, leading to a significant reduction in SiNPs-induced COX-2 protein levels (Figure 5C). To explore the necessity of PKCα phosphorylation in SiNPs-triggered responses, we analyzed phospho-PKCα levels through Western blot. SiNPs progressively enhanced PKCα phosphorylation, peaking from 60–180 min, a response significantly mitigated by Gö6976 or PF431396 treatment. Notably, Gö6976 treatment had no significant impact on SiNPs-stimulated Pyk2 phosphorylation, indicating that Pyk2 acted as an upstream component of PKCα in SiNPs-mediated responses (Figure 5D). Moreover, treatment with PF431396 attenuated SiNPs-induced PGE_2_ secretion in HTSMCs (Figure 5E). These findings strongly indicate that PKCα mediates SiNPs-induced COX-2 expression in HTSMCs, with Pyk2 serving as an upstream regulator of PKCα activation in this process.

### 3.6. The p42/p44 MAPK Pathway Is Involved in SiNPs-Induced COX-2 Expression and PGE_2_ Synthesis

**Figure 6:**
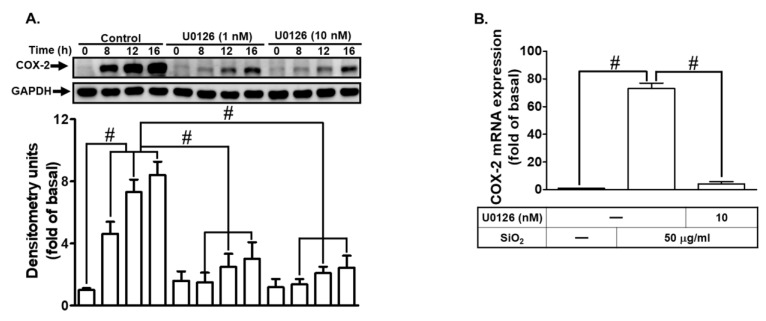

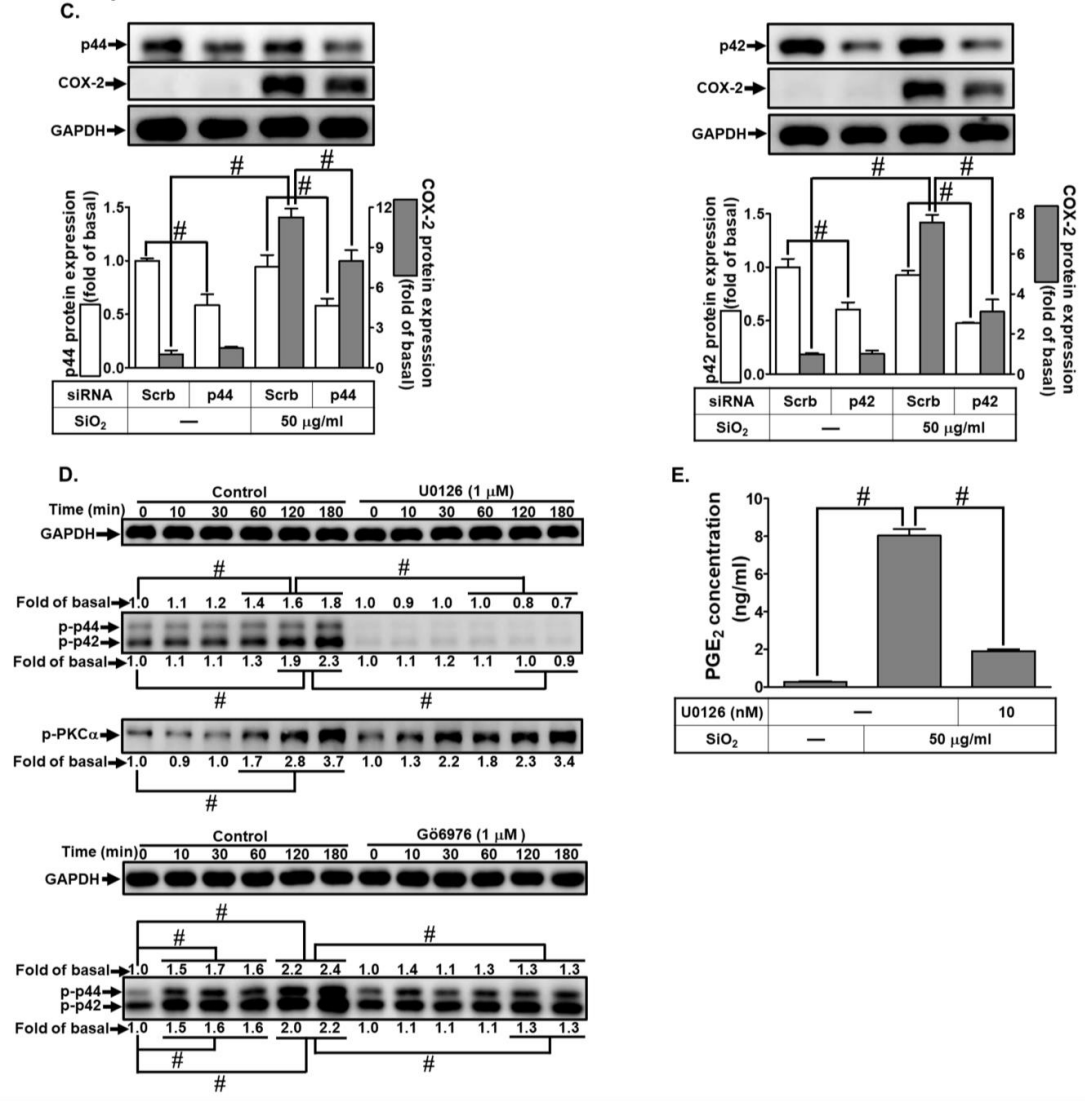
The p42/p44 MAPK participates in SiNPs-induced COX-2 expression and PGE_2_ synthesis in HTSMCs. (**A**) Cells were pre-incubated with U0126 (0, 1, and 10 nM) for 1 h and then exposed to SiNPs (50 μg/mL) for 0, 8, 12, and 16 h. Western blot was conducted to assess the expression of COX-2, with GAPDH serving as an internal control. (**B**) Cells were pre-treated with U0126 (10 nM) for 1 h and subsequently incubated with SiNPs (50 μg/mL) for 4 h. Total RNA was extracted and analyzed by real-time PCR. (**C**) Cells were transfected with PKCα siRNA and then treated with SiNPs (50 μg/mL) for 16 h. Western blot was performed to evaluate the expression of total PKCα, COX-2, and GAPDH. (**D**) Cells were pre-treated with U0126 (1 μM) or Gö6976 (1 μM) for 1 h and then incubated with SiNPs for the specified time intervals. Western blot was used to analyze the expression of phosphorylated p42/p44 MAPK and PKCα. (**E**) Cells were pre-treated with U0126 (10 nM) for 1 h and then exposed to SiNPs (50 μg/mL) for 16 h. The culture medium was collected and analyzed using ELISA to measure PGE_2_ secretion. The data are represented as mean ± S.E.M. from three separate experiments. # *p* < 0.05 indicates a significant difference between the groups.

A study by Yan et al. [22] demonstrated that exposure to combustion-derived metal Zn^2+^ activates MAPKs (JNK1/2, p38 MAPK, p42/p44 MAPK), inducing IL-8 and COX-2 expression in airway cell lines. To probe the role of p42/p44 MAPK in SiNPs-exposed HTSMCs, we utilized the MEK1/2 inhibitor U0126. Treatment with U0126 significantly dampened SiNPs-induced COX-2 protein and mRNA expression (Figure 6A,B). To further elucidate the involvement of p42/p44 MAPK in SiNPs-induced COX-2 expression, we employed p42 or p44 siRNA transfection, resulting in the knockdown of total p42 or p44 protein expression. Consequently, COX-2 expression was abolished in SiNPs-challenged HTSMCs (Figure 6C). Additionally, SiNPs triggered a time-dependent phosphorylation of p42/p44 MAPK, a response attenuated by U0126 and Gö6976. U0126 treatment had no significant impact on SiNPs-induced PKCα phosphorylation, indicating that PKCα acted as an upstream regulator of p42/p44 MAPK in SiNPs-mediated responses (Figure 6D). Furthermore, treatment with U0126 reduced SiNPs-induced PGE_2_ secretion (Figure 6E). In summary, our findings suggest that SiNPs-induced COX-2 expression in HTSMCs occurs through the PKCα- and p42/p44 MAPK-dependent pathway.

### 3.7. The Involvement of NF-κB in SiNPs-Induced COX-2 Expression and PGE_2_ Synthesis

**Figure 7:**
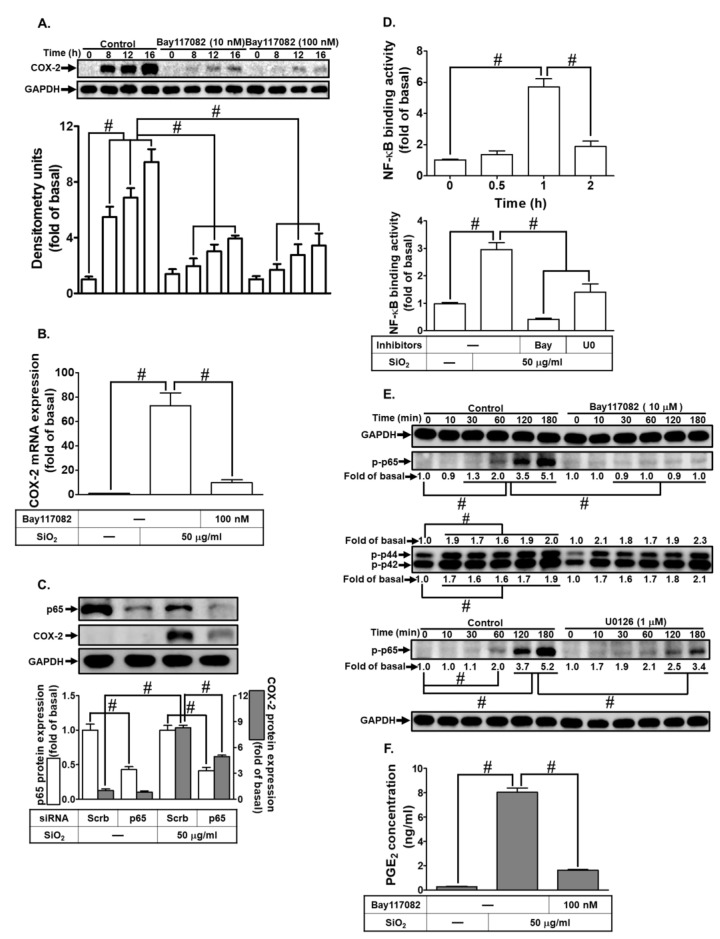
Influence of NF-κB on SiNPs-induced COX-2 expression and PGE_2_ synthesis in HTSMCs. (**A**) Cells were pre-treated with the NF-κB inhibitor, Bay117082 (100 nM), for 1 h and then exposed to SiNPs (50 μg/mL) for 0, 8, 12, and 16 h. Western blot was conducted to assess the expression of COX-2 and GAPDH. (**B**) Cells were pre-treated with Bay117082 (100 nM) for 1 h and subsequently incubated with SiNPs (50 μg/mL) for 4 h. Total RNAs were harvested and analyzed by real-time PCR. (**C**) Cells were transfected with p65 siRNA and then treated with SiNPs (50 μg/mL) for 16 h. Western blot was performed to evaluate the expression of total p65, COX-2, and GAPDH. (**D**) Cells were treated with SiNPs for the specified time interval or pre-treated with Bay117082 (100 nM) for 1 h and then incubated with SiNPs (50 μg/mL) for 1 h. The ChIP assay was conducted to analyze NF-κB p65 binding to DNA. (**E**) Cells were pre-treated with Bay117082 (10 μM) or U0126 (1 μM) for 1 h, followed by incubation with SiNPs for the indicated time interval. Western blot was used to analyze the expression of phosphorylated p65 and p42/p44 MAPK. (**F**) Cells were pre-treated with Bay117082 (100 nM) for 1 h and then exposed to SiNPs (50 μg/mL) for 16 h. The culture medium was collected and analyzed by ELISA to examine PGE_2_ secretion. The data are represented as mean ± S.E.M. from three separate experiments. # *p* < 0.05 indicates a significant difference between the groups.

To explore the impact of NF-κB on COX-2-mediated PGE_2_ production, we utilized the NF-κB inhibitor, Bay117082. As shown in Figure 7A,B, SiNPs-induced COX-2 protein and mRNA expression were significantly reduced by Bay117082. To delve deeper into the involvement of NF-κB p65, we used p65 siRNA transfection, leading to a knockdown of total p65 protein expression and subsequent abolition of COX-2 expression in HTSMCs (Figure 7C). To investigate the regulatory mechanisms of NF-κB p65 activity on the COX-2 promoter region, we conducted a ChIP assay. Stimulation of HTSMCs with SiNPs led to a time-dependent increase in NF-κB p65 binding to the COX-2 promoter region, with a significant binding response observed within 1 h. Furthermore, this p65 binding was inhibited by treatment with Bay117082 or U0126 (Figure 7D). Consistent with these findings, SiNPs-induced phosphorylation of p65 was observed, a response suppressed by Bay117082 or U0126 treatment. Notably, Bay117082 treatment did not significantly affect SiNPs-stimulated p42/p44 MAPK phosphorylation, suggesting that NF-κB p65 operates as a downstream component of p42/p44 MAPK in SiNPs-mediated responses (Figure 7E). Finally, we showed that pre-treatment with Bay117082 reduced SiNPs-induced PGE_2_ secretion (Figure 7F). These results indicate the involvement of the NF-κB p65 transcription factor in SiNPs-induced COX-2 expression, with activation occurring downstream of p42/p44 MAPK signaling.

## 4. Discussion

**Figure 8:**
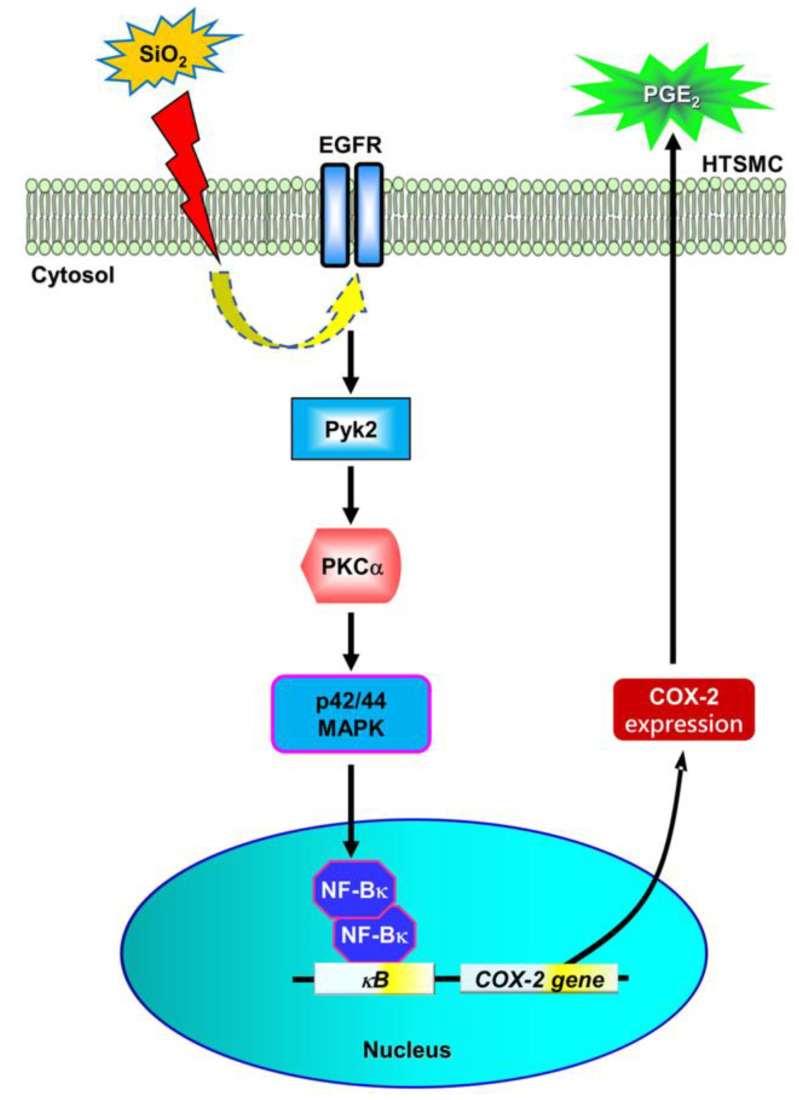
A schematic diagram proposing the mechanism of SiNPs-induced COX-2 expression and PGE_2_ synthesis in HTSMCs. Upon exposure to SiNPs, the process initiates with the activation of EGFR. This activation triggers a cascade involving Pyk2, PKCα, and p42/p44 MAPK, ultimately leading to the activation of NF-κB. The activated NF-κB p65 translocates to the cell nucleus and binds to the promoter region of the COX-2 gene. This binding event promotes the transcription of COX-2, subsequently enhancing PGE_2_ synthesis and production in HTSMCs. This proposed mechanism sheds light on the intricate molecular pathways through which SiNPs induce the expression of COX-2 and drive PGE_2_ synthesis in HTSMCs, providing valuable insights into the cellular response to nanoparticle exposure.

Understanding the potential risks associated with the widespread use of SiNPs, particularly in the food industry, is crucial for mitigating the potential health hazards, including pulmonary and neurodegenerative disorders [16]. In our study, the exposure of HTSMCs to SiNPs led to a significant increase in COX-2 expression and the release of PGE_2_ autacoid. Considering the pivotal role of PGE_2_ in regulating various physiological activities and acting as a pro-inflammatory mediator in respiratory diseases such as COPD [26], it is imperative to comprehend the potential impact of SiNPs on airway smooth muscle cells. This understanding is essential as it could interfere with the contractility and physiological functions of tracheal smooth muscle in vivo. The signaling cascade triggered in SiNPs-treated HTSMCs can be summarized as follows (Figure 8). First and foremost, our results indicated that COX-2 expression in SiNPs-treated HTSMCs was abolished by the EGFR inhibitor (AG1478) and EGFR siRNA, suggesting that EGFR mediates SiNPs-induced COX-2 expression. Second, the downstream components of EGFR signaling responsible for this induction involve Pyk2, PKCα, p42/44 MAPK, and NF-κB. These findings are consistent with previous research linking EGFR-dependent signaling to the pathophysiology of asthma, an airway disease [27]. In conclusion, unraveling the intricate signaling pathways activated in response to SiNPs in HTSMCs is not only essential for understanding the molecular mechanisms underlying their toxicity but also critical for developing strategies to mitigate their adverse effects on respiratory health.

The involvement of Pyk2 phosphorylation in SiNPs-treated HTSMCs is a compelling aspect of our study. Our research demonstrated that SiNPs treatment triggered Pyk2 phosphorylation, a phenomenon that was effectively suppressed by AG1478 (EGFR inhibitor) treatment. Additionally, we found that inhibiting Pyk2 phosphorylation, either through the use of PF431396 (a Pyk2 inhibitor) or siRNA interference, led to the abolishment of SiNPs-induced COX-2 expression and PGE_2_ production. This implicates a pivotal role of Pyk2 in the cellular responses to SiNPs. Notably, Pyk2 has previously been linked to superoxide release [24] and is highly expressed in acute lung injury conditions [28]. This association suggests a potential connection between Pyk2 and NADPH oxidase/ROS signaling pathways in the context of lung inflammation. It is plausible that NADPH oxidase/ROS activation precedes Pyk2 activation in response to certain stimuli. While this hypothesis was not further explored in our current study, our findings clearly demonstrate that SiNPs induce COX-2 expression through the EGFR and Pyk2 pathways in HTSMCs. This discovery sheds light on the intricate interplay between SiNPs, EGFR, Pyk2, and inflammatory responses in HTSMCs. Understanding the detailed molecular mechanisms underlying these interactions could potentially provide valuable insights into the development of targeted therapies for lung inflammation associated with nanoparticle exposure. Further investigations are warranted to unravel the specific cascade of events leading to Pyk2 phosphorylation and its subsequent impact on NADPH oxidase/ROS-mediated inflammatory pathways in lung cells.

In the realm of cellular signaling, PKC enzymes, crucial mediators in various biological processes, exhibit distinctive activation profiles. As delineated by Dempsey et al. [25], PKCs are categorized into three classes: classical (cPKC), novel (nPKC), and atypical (aPKC). Classical PKCs (α, βI, βII, and γ) respond to both calcium ions (Ca^2+^) and diacylglycerol (DAG), while novel PKCs (δ, ε, η, and θ) are solely activated by DAG, independent of Ca^2+^. Atypical PKCs (λ, ι, and ζ) stand apart, being insensitive to both Ca^2+^ and DAG. Additionally, a unique class represented by PKCμ, also known as PKD, responds to DAG but operates autonomously of Ca^2+^ [29]. A significant revelation emerged concerning the impact of SiNPs on PKCα phosphorylation. This phenomenon was substantiated by experiments where the specific inhibition of PKCα using Gö6976 and siRNA-mediated knockdown of PKCα influenced SiNPs-induced COX-2 expression and PGE_2_ production in HTSMCs. These results unequivocally suggest the involvement of PKCα in SiNPs-induced COX-2 expression and PGE_2_ production in HTSMCs. Intriguingly, a similar effect of SiNPs on murine GC-2 spermatocytes was previously reported. In this context, SiNPs were found to activate the PKCδ/p53/p21^Cip1^ and PKCα/MAPK signaling pathways [30]. The intricate web of PKC activation is not limited to specific stimuli; PKCs can be triggered by diverse cellular events, including the activation of RTKs, G protein-coupled receptors (GPCRs), and integrins [31]. Notably, there exists a plethora of RTKs in the human cellular milieu, with approximately 58 well-characterized members, many of which orchestrate the activation of PKCs [32]. In the current study, inhibition of non-RTK Pyk2, using PF431396, resulted in compromised PKCα phosphorylation in HTSMCs. Building on our prior understanding that Pyk2 becomes activated subsequent to EGFR activation, we postulate a sequential activation cascade wherein SiNPs stimulate COX-2 expression through the EGFR/PYK2/PKCα axis in HTSMCs. This intricate interplay between SiNPs and cellular signaling pathways sheds light on the nuanced mechanisms underpinning SiNPs-induced responses in diverse cell types, emphasizing the multifaceted nature of nanoparticle–cell interactions.

In the intricate landscape of inflammatory responses, cells stimulated with PM activate diverse signaling pathways such as MAPKs, PI3K/Akt, and TLRs, underscoring the complexity of cellular reactions to environmental challenges [33]. Previous studies have illuminated the impact of crystalline silica on cellular responses. For instance, crystalline silica induces COX-2 expression in lung A549 epithelial cells, accompanied by the phosphorylation of p42/p44 MAPK and p38 MAPK, while the involvement of p42/p44 MAPK and p38 MAPK pathways in AP-1 induction has been demonstrated in murine epidermal JB6 cells [34,35]. Interestingly, SV-40-transformed human bronchial epithelial BEAS-2B cells exhibit significant phosphorylation of both p38 MAPK and JNK1/2 in response to silica challenge, indicating the existence of cell-type-specific variations in MAPK activation [21]. In our current investigation, SiNPs-induced phosphorylation of p42/p44 MAPK was effectively inhibited by U0126 and the PKCα/β inhibitor, Gö6976, in HTSMCs. This observation suggests a hierarchical activation sequence wherein p42/p44 MAPK is downstream of PKCα in SiNPs-challenged HTSMCs. Moreover, the indispensability of NF-κB in SiNPs-induced COX-2 expression and PGE_2_ production was established through experiments utilizing BAY117082 and NF-κB p65 siRNA, corroborating the pivotal role of NF-κB signaling in this context. This intricate signaling network finds its basis in the regulatory regions of the COX-2 gene. Analysis of the 5′-flanking region of the COX-2 promoter has revealed the presence of multiple binding sites for pivotal transcription factors, including AP-1 and NF-κB, underscoring the intricate transcriptional regulation of COX-2 expression [36].

In summary, we have unearthed significant insights into the intricate molecular mechanisms of SiNPs on HTSMCs. We discovered that SiNPs initiate the expression of COX-2 and the synthesis of PGE_2_, a process that is distinctly time- and concentration-dependent. Further exploration revealed that SiNPs induce COX-2 expression and PGE_2_ release in HTSMCs through the activation of a complex signaling network involving the EGFR, Pyk2, PKCα, and the p42/p44 MAPK pathway, culminating in NF-κB signaling (Figure 8). This response to SiNPs is significant, not only for its immediate impact on cellular functions but also for its potential implications in respiratory health. The elevated production of PGE_2_ in response to SiNPs exposure could be a compensatory mechanism or a detrimental factor, given its dual role in mediating bronchodilation and inflammation. This duality presents a therapeutic opportunity, where targeting proteins like COX-2 and their downstream signaling components could be key in mitigating airway injuries and inflammatory diseases triggered by SiNPs.

## 5. Conclusions

In conclusion, our research provides a comprehensive understanding of the molecular responses induced by SiNPs in HTSMCs, delineating a complex signaling cascade that leads to significant biological effects. These findings not only highlight the critical role of SiNPs in modulating cellular functions but also open up new avenues for biomedical research and therapeutic interventions. By revealing the nuanced interactions within the EGFR/Pyk2/PKCα/p42/p44 MAPK-dependent NF-κB signaling pathway and their implications in COX-2 expression and PGE_2_ secretion, our study lays the groundwork for future explorations into novel therapeutic targets. This could significantly advance our understanding of nanoparticle-induced cellular responses and their potential impact on respiratory health. Our research paves the way for future studies to further explore the therapeutic potential and safety of SiNPs in biomedical applications, particularly in the context of respiratory diseases.

## Data Availability

The data presented in this study are available on request from the corresponding author.
